# Integrated syphilis/HIV screening in China: a qualitative analysis

**DOI:** 10.1186/1472-6963-10-58

**Published:** 2010-03-07

**Authors:** Joseph D Tucker, Li-Gang Yang, Zheng-Jun Zhu, Bin Yang, Yue-Pin Yin, Myron S Cohen, Xiang-Sheng Chen

**Affiliations:** 1 Division of Infectious Diseases, Massachusetts General Hospital, Boston, USA; 2 Guangdong Provincial Center for STI Control & Prevention, Guangzhou, China; 3 Jiangmen Skin Hospital, Jiangmen, China; 4 National Center for STD Control, Nanjing, China; 5 Institute for Global Health and Infectious Diseases, UNC Chapel Hill School of Medicine, Chapel Hill, USA

## Abstract

**Background:**

The last decade has seen enormous advances in HIV treatment and care, but how to implement scaled up HIV testing, prevention, and treatment in low-income areas still presents a formidable public health challenge. South China faces expanding syphilis and sexually transmitted HIV epidemics, but health systems characteristics important for scaling up syphilis and HIV testing have not been defined.

**Methods:**

A purposive sample to ensure public, private, and public-private hybrid STI clinic inclusion was selected in a South China city. Eight key informant interviews were conducted with the STI clinic manager, followed by eight focus group discussions with physicians. Data collection relied on a semi-structured format that included questions in each of the following domains: 1) clinical facilities; 2) laboratory capacity with a focus on syphilis/HIV diagnosis; 3) clinic personnel; 4) physical space with a focus on locations to disclose confidential results; 5) financial support.

**Results:**

Public STI clinics had free syphilis testing/treatment and laboratory facilities to perform essential syphilis and HIV tests. However, despite serving a large number of STI patients, private STI clinics lacked nontreponemal syphilis testing, HIV testing, and had fewer connections to the public health infrastructure. Formally trained assistant physicians were 2.5 times as common as physicians at STI clinics. Only one of the 8 sites had onsite voluntary counseling and testing (VCT) services available.

**Conclusion:**

These STI case studies reveal the potential for expanding integrated syphilis/HIV services at public STI clinics in China. More health services research is needed to guide scale-up of syphilis/HIV testing in China.

## Background

The last decade has seen enormous advances in HIV treatment and care, but how to implement scaled up HIV testing, prevention, and treatment in low-income areas still presents a formidable public health challenge [1]. Approximately 90% of new syphilis cases globally are in low-income areas where sexually transmitted HIV is also a major public health problem [2]. Syphilis increases the risk of HIV acquisition [3] and transmission [4,5] and patients with either STI frequently attend the same STI clinics. In 2008, China had 278,215 officially reported syphilis cases, a threefold rise in the number of reported cases compared to 2004 and a tenfold increase over the last decade [6,7]. Effective scale-up of integrated syphilis/HIV services demands scalable, high quality clinical STI services.

While the need for integrated syphilis/HIV testing at STI clinics in China has increased, health market reforms in China have limited the capacity of local STI clinics to meet the growing demand for syphilis/HIV screening services [8]. The army of barefoot doctors in the context of a broad reaching anti-epidemic disease station system subsidized by the state has largely been replaced by a fee-for-service STI clinic system. Although China has a nationwide network of standardized public STI clinics, this established infrastructure has been bypassed by large numbers of STI patients who seek care at private clinics [9-14]. The literature on private STI clinics, including syphilis/HIV testing capacity and human resources, is scant [15].

**Figure 1:**
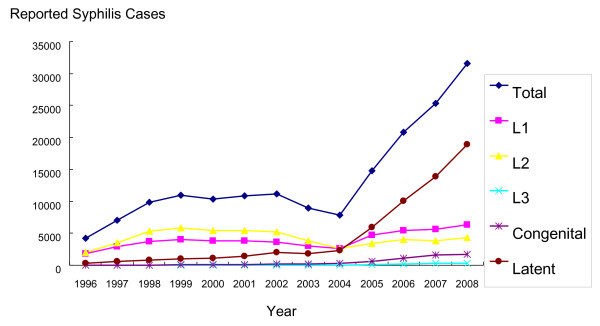
**Reported syphilis cases in Guangdong Province, China, from 1996-2008**. L1 -- primary syphilis; L2 -- secondary syphilis; L3 -- tertiary syphilis. Source: Guangdong Provincial STI/Leprosy Conference 2009, Jiangmen City, China.

In order to understand the scalability of providing integrated syphilis/HIV services at STI clinics in China, we conducted operational STI clinic research in Guangdong Province, a rapidly developing region with increasing syphilis burden in South China (Figure 1). Public STI clinics were selected instead of other clinical facilities that might provide similar syphilis/HIV services (obstetrics and gynecology, urology, family planning, etc) because STI clinics have a higher burden of STIs [12] and a national infrastructure that reaches to the township level. These case studies analyzed the clinical, laboratory, and human capacity at various types of STI clinics in China to inform implementation of integrated syphilis/HIV testing efforts and guide scale-up policy. This research employed qualitative methods because little is known about the operational characteristics of STI clinics, especially non-public clinics, in China.

## Methods

Jiangmen City is located in the southcentral region of Guangdong Province, west of the Pearl River Delta. Adjacent to Zhongshan in the east and Foshan in the north, the city faces southward to the South Sea and neighbors Hong Kong and Macau. It is accessible to Hong Kong via a two-hour ferry ride, and has major highways that connect to the provincial capital, Guangzhou, and Hong Kong. Jiangmen has a total population of 3.9 million, and consists of 3 districts and 4 county-level cities.

A qualitative approach was used because of the limited data available to structure quantitative data collection [12] and the complexity of social factors in relation to operational clinic characteristics [16]. The formative data from this qualitative study then informed a larger quantitative analysis of syphilis/HIV testing at STI clinics. Interviews with STI clinic managers were chosen since managers have first-hand knowledge of many operational variables unknown to individual physicians (e.g., laboratory grade). Focus groups were also used with physicians because of convenience and the limited sensitive material, consistent with other studies focused on operational characteristics of health care in China [17,18]. A purposive technique was used to select a convenience sample of STI clinics in Jiangmen City in order to ensure a range of clinic types, including public, private, and public-private hybrid STI clinics. A total of eight STI clinics were chosen, and each of the clinics was contacted by telephone to review study eligibility.

An interview guide was assembled based on the following core domains related to delivery of integrated syphilis/HIV testing: a) clinical facilities (e.g., daily number of patients) b) laboratory capacity with a focus on syphilis/HIV testing (tests available onsite and referred away); c) clinic personnel; d) physical space with a focus on locations to disclose confidential results; e) financial support. Domains were chosen based on their relevance to implementation of syphilis/HIV testing services [19,20]. Survey items used previously in China to evaluate HIV testing among physicians [21] were expanded to include syphilis testing. All survey items were pre-tested with physicians in the public health bureau prior to data collection. Standardized Chinese public health grades were used to classify laboratory capacity, ranging from 1 (most basic, able to perform rapid test) to 3 (most advanced, able to perform HIV CD4 counts). Key informant interviews with STI clinic managers were conducted in a private, enclosed room away from clinical services. The interviews were conducted one-on-one with a trained research assistant in the local language, and notes were recorded by two research assistants. After key informant interviews, STI clinic managers gave a tour of the physical facilities with a focus on individual clinic rooms and clinic laboratory facilities. STI clinic manager meetings preceded the focus groups and this ordering was used to contextualize and further refine appropriate questions for focus group discussions. Focus group discussions included clinicians, laboratory technicians, and nurses. All research notes were transcribed and translated into English, and open-ended questions were coded by themes and analyzed by two independent research assistants. Conflicts in the data were resolved by the lead investigator and a summary data were produced for each of the eight study sites based on the results of coding. This study was approved by the National STD Center IRB, the UNC IRB, and the Partners Human Research Committee.

## Results

Among the eight STI clinics included in this study, three were independent public clinics, three were general hospital-associated, one was completely private, and one was a public/private hybrid (Table 1). The three independent STI clinics were administratively underneath the national STI control center within the Chinese Centers for Disease Control while the public general hospital STI clinics were administratively within the general hospital infrastructure. These two systems both report syphilis and HIV cases to the local public health authorities and have similar clinical responsibilities, although there was limited communication, shared training, or other joint activities. Seven out of eight of the clinics perceived private clinics as the main competitor in terms of providing local STI services. There were large variations in the estimated nonunique STI patients per year (Table 1). Neither of the private STI clinics had formal laboratories that could be rated using established laboratory certification criteria, but five of six public clinics had laboratories that met Chinese certification criteria (Table 1). All clinics reported short patient waiting times, generally less than 30 minutes to be evaluated by a physician.

**Table 1:** Operational characteristics of eight STI clinics in South China

#	Type	STI visits per year	Total personnel	MD^1^/assistant MD/RN	Certified Laboratory^2^	Hours	CDC link	Gov't support
1	Public independent clinic	~3,900	60	3/14/22	Grade 2	8 am -- 6:30 pm	Yes	Yes
2	Public independent clinic	~940 - 1,560	20	0/5/6	Informal	8:00 am -- 6:30 pm	Yes	Yes
3	Public independent clinic	~1450	30-35	2/5/5	Grade 2	9:00 am -- 6:00 pm	Yes	Yes
4	Public general hospital	~610 - 930	4	3/0/1	Grade 3	8:00 am -- 5:30 pm	Yes	Yes
5	Public general hospital	~260 - 520	2	0/2/1	Grade 3	8:00 am -- 5:30 pm	No	Yes
6	Public general hospital	~5720 - 8580	6	2/2/1	Grade 3	8:00 am -- 5:00 pm	Yes	Yes
7	Private	~1090 - 1820	35	5/6/15	Informal	24 hours	No	No
8	Public/private^3^	~10,600	100	10/30/35	Informal	8:00 am -- 9:30 pm	Refer patients to CDC ID	Yes^3^

^1 ^Definitions of health care providers used standard Chinese medical classifications [31].

^2 ^China's system of laboratory specifications has three tiers -- grade 3 (highest level), grade 2 (mid level), grade 1 (lowest level).

^3 ^This is a private clinic that operates with limited government support in direct relationship to the amount of money that the clinic pays.

Our data revealed substantial variation in medical training backgrounds. Licensed physicians in China have at least a bachelor's degree in medicine, pass a licensure examination, and complete one year of supervised internship at a clinic or preventive health institution. Licensed assistant physicians must have at least two years of medical training at a technical school following high school, pass a licensure examination, and complete one year of supervised internship at a clinic or preventive health institution. Nurses must have at least two years of training following high school, but no internship or examination is required. Across all eight sites, 28% of the STI clinicians were licensed physicians and 72% of STI clinicians were licensed assistant physicians, representing a 2.5-fold difference. The total number of nurses at all clinics (86) was similar to the number of physicians (89).

Rapid treponemal syphilis testing was available in both the central public STI clinics and private clinics, but supported by different payment mechanisms (Table 2). The central public clinics were supported by a World Health Organization Sexually Transmitted Diseases Initiative to pay for syphilis rapid testing while the two private clinics charged patients for rapid syphilis testing. The turnaround times reported for RPR tests were quite minimal, generally less than one day. Seven out of eight STI clinics had private rooms for the patient-physician encounter, but one of the general hospital-associated clinics had shared rooms for clinical STI evaluation.

**Table 2:** HIV testing, syphilis testing, and related public health capacity at 8 STI clinics in South China

#	Syphilis Nontreponem Test/price/turnaround time	Syphilis Treponemal Test	Free syphilis treatment	HIV ELISA/price/turnaround time	One-on-one rooms	Counseling	Reporting to official system	Referral of HIV+ to local CDC
1	RPR/free/several hours	Rapid test/free/minutes	Yes	Yes/$9/same day	Yes	Free VCT	Yes	Yes
2	RPR/free/several hours	Rapid test/free/minutes	Yes	Yes/$11/same day	Yes	None	Yes	Yes
3	RPR/$7/several hours	TPPA/$5/several hours	No	Yes/$10/several hours	Yes	None	Yes	Yes
4	RPR/$5/3 days	TPPA/$3/3 days	No	Yes/$9/3 days	No	None	Yes	Yes
5	RPR/TPPA combined $7/several hours	No	Yes/$9/hours	Yes	Yes	Yes	Yes	
6	RPR/$1.50/30 minutes	TPPA/$10/hours	No	Yes/$9/hours	Yes	None	Yes	Yes
7	none	Rapid test/$5/few minutes	No	Yes/$9/minutes	Yes	None	No	Yes
8	none	TRUST/$1.50/minutes	No	No	Yes	Limited on the phone	Yes	NA

Abbreviations - RPR, rapid plasma reagin; TPPA, Treponema Pallidum particle agglutination; Treponema Pallidum haemagglutination test. FTA-Abs, fluorescent treponemal antibody absorbed; VDRL, Venereal Disease Research Laboratory.

Clinical STI service funding varied substantially according to clinic type. Public and hybrid STI clinics received direct support from the government and patient fees while the cost of private clinics is supported entirely by patient fees. Relationships with pharmaceutical representatives ranged from an explicit policy of no pharmaceutical representatives in the clinical area to pharmaceutical representatives in the same room as the physician and patient during the clinical encounter. Information on how ordering tests affected physician salaries was incomplete, but one private staff physician reported that there were direct financial incentives to order tests. This STI testing incentive did not apply to "free" tests provided by the STI clinic since these did not impact the clinic revenue or their salary.

## Discussion

This study provides the first English language operational research on STI clinics in China, one of the key factors involved in scaling up high quality syphilis/HIV services. The large number of assistant physicians and nurses in STI clinics at both public and private STI clinics is important since training background has been shown to be associated with HIV stigma in China [22]. Inadequate HIV knowledge and high levels of HIV stigma have been observed among nurses and nursing students in China [22-24].

The finding that public STI clinics have greater laboratory capacity [25,26], more trained personnel [27], and more clear reporting to the public health infrastructure [28] has been observed outside of China. The correlation between STI physician salaries and revenues generated from testing has also been described elsewhere. A US study found that privatized STI clinics were more likely to order tests for *C. trachomatis *and *N. gonorrhoeae*, both of which were reimbursed, rather than HIV and syphilis serologies [25]. This connection between physician salaries and diagnostic tests could have important implications for scaling up syphilis/HIV testing programs. The use of rapid syphilis and HIV tests at private clinics in China is surprising given that private clinicians are less likely to use rapid HIV tests in other contexts [28,29].

The substantial reported volume of private STI clinic patients is supported by several Chinese studies. Among those who reported getting treatment for an STD in the national 2000 census, 38% received treatment in private clinics and 25% never presented to a formal clinic [10]. One study found from Western China found that only 41% of STI patients went directly to a public clinic first, and where the patient lived was significantly related to their choice of clinic [11]. A study of STI/HIV care in Guangzhou and Shenzhen found that among 939 individuals, delays in receiving treatment among men were associated with seeking care at a private STI clinic [15]. In a study of health seeking behaviors from a large district of Beijing, only 2% of the sample sought care from a public STI clinic, while 61% sought care from other sources (private clinics, hospitals, and pharmacies)[30]. These studies confirm the importance of the private STI clinic in serving the needs of high risk populations in China.

This study has several limitations that merit discussion. Jiangmen City has greater economic resources than many cities in China, so the results cannot be generalized to all regions of China. In addition, each case study selected to investigate is part of a convenience sample that does not represent all STI clinic typologies. This limitation is perhaps most pronounced in the case of the private clinics where the illegal, underground nature of some private STI clinics makes identification of both clinics and a sampling unit challenging. Unlicensed physicians also provide STI services in China, but were not sampled in this study. In addition, accurately recording physician reported information about reimbursement and financial incentives can be a sensitive issue. Although this research used both one-on-one interviews and small group interviews, using longer in-depth interviews may have uncovered more nuanced attitudes and behaviors related to financial incentives.

## Conclusions

STI clinics in China are centrally important to responding to the related epidemics of syphilis and HIV infection. Our qualitative analysis of STI clinic physicians and managers in Guangdong Province found that public STI clinics had greater laboratory capacity, human resources, and connections to the public health system. The large number of assistant physicians at STI clinics in China can serve important roles in implementation of integrated syphilis and HIV testing. More health services research is needed about STI clinic systems in China to inform scale-up of effective syphilis/HIV testing programs.

## Competing interests

The authors declare that they have no competing interests.

## Authors' contributions

All authors were involved in the conception and design of the study; JT, LY, MC, and XC analyzed and interpreted the data; all authors except MC helped draft the paper or substantially revised it. All the authors read and approved the final manuscript.

## Pre-publication history

The pre-publication history for this paper can be accessed here:

http://www.biomedcentral.com/1472-6963/10/58/prepub
